# *Saccharomyces cerevisiae* CNCM I-3856 as a New Therapeutic Agent Against Oropharyngeal Candidiasis

**DOI:** 10.3389/fmicb.2019.01469

**Published:** 2019-07-11

**Authors:** Elena Roselletti, Samuele Sabbatini, Nathalie Ballet, Stefano Perito, Eva Pericolini, Elisabetta Blasi, Paolo Mosci, Amélie Cayzeele Decherf, Claudia Monari, Anna Vecchiarelli

**Affiliations:** ^1^ Medical Microbiology Section, Department of Medicine, University of Perugia, Perugia, Italy; ^2^ Lesaffre International, Lesaffre Group, Marcq-en-Baroeul, France; ^3^ Department of Surgical, Medical, Dental and Morphological Sciences With Interest in Transplant, Oncological and Regenerative Medicine, University of Modena and Reggio Emilia, Modena, Italy; ^4^ Internal Medicine Section, Department of Veterinary Medicine, University of Perugia, Perugia, Italy; ^5^ Gnosis by Lesaffre, Lesaffre Group, Marcq-en-Baroeul, France

**Keywords:** oropharyngeal candidiasis, oral infections, probiotics, *Saccharomyces cerevisiae*, *Candida albicans*, yeasts

## Abstract

Oropharyngeal candidiasis is a common opportunistic mucosal infection of the oral cavity, mainly caused by an overgrowth of *Candida albicans*. This infection can inhibit nutritional intakes and strongly affect quality of life. To date, standard therapeutic strategies involving the administration of antifungal drugs can bring several side effects, not least the emergence of drug-resistant strains. The purpose of this study is to investigate the effectiveness of *Saccharomyces cerevisiae* CNCM I-3856 (live or inactivated cells) against oropharyngeal candidiasis. Our results show that administration of *S. cerevisiae* CNCM I-3856 (live or inactivated cells) in the oral cavity of C57BL/6J mice resulted in a protective effect against oropharyngeal candidiasis. The strongest effect was obtained with live *S. cerevisiae* CNCM I-3856. This was related to: (1) a decrease in *C. albicans* load in the oral cavity, esophagus, stomach, and duodenum; (2) an early resolution of inflammatory process in the tongue; (3) a marked reduction in *C. albicans* virulence factors; and (4) a consistent increase in neutrophil antimicrobial capacity. These findings suggest that *S. cerevisiae* products are potentially beneficial in the treatment of oropharyngeal candidiasis.

## Introduction

*Candida albicans* is an opportunistic pathogenic fungus that commonly inhabits the mouth, vagina, and intestinal tract of healthy individuals, causing many different types of mucosal infections, including oral candidiasis. Also known as thrush or oropharyngeal candidiasis (OPC), oral candidiasis is the most common opportunistic infection of the oral cavity (Akpan and Morgan, 2002; Gonzalez Gravina et al., 2007; Naglik and Moyes, 2011; Melkoumov et al., 2013; Berberi et al., 2015; Kenno et al., 2016). It is especially common and underdiagnosed among the elderly (particularly in those wearing dentures), infants, and immunocompromised individuals or individuals on long-term antibiotic treatments. It can also be linked to systemic diseases such as diabetes mellitus. In individuals with defective immune response, the pathogen may spread to the pharynx and the esophagus, causing severe symptoms such as erosions and ulcerations of the tissues. In addition, it spreads through the gastrointestinal tract, predisposing its host to the development of systemic or disseminated candidiasis, leading to high morbidity and mortality rates (Gudlaugsson et al., 2003; Pappas, 2006). Indeed, compelling evidence shows that the gastrointestinal tract could be the largest source of candidemia when systemic or local mucosal immune functions are disturbed (Li et al., 2000; Miranda et al., 2009; Schulze and Sonnenborn, 2009). To date, antifungal drug administration (nystatin, amphotericin B, fluconazole (FLZ), itraconazole, and voriconazole) represents the first line therapy against candidiasis (Garcia-Cuesta et al., 2014). However, the numerous side effects (Ohshima et al., 2016) induced by drugs toxicity (Sardi et al., 2013) and the appearance of drug-resistant strains (Matsubara et al., 2016; Ohshima et al., 2016) emphasize the urgency of developing innovative therapeutic strategies. In this regard, probiotics can represent a promising alternative approach. Several studies have reported the anti-pathogenic potential of probiotic bacteria for the prevention and/or the treatment of oropharyngeal candidiasis (Hatakka et al., 2007; Ishijima et al., 2012; Ishikawa et al., 2015; Kraft-Bodi et al., 2015; Matsubara et al., 2016; Ohshima et al., 2016). Recently, Leão et al. (2018) observed that the presence of *Lactobacillus rhamnosus* ATCC 7469, prior to inoculation with *C. albicans* clinical strain, avoided the colonization and consequently the growth of the pathogen, thus effectively preventing the development of candidiasis in immunosuppressed mice. To our knowledge, only one study supported the efficacy of *Saccharomyces cerevisiae* treatment by local application (Premanathan et al., 2011). Unfortunately, the *S. cerevisiae* strain used in this study was not defined. We recently demonstrated that live and inactivated *S. cerevisiae* CNCM I-3856 show a therapeutic activity in an experimental model of vaginal candidiasis in mice (Pericolini et al., 2017; Gabrielli et al., 2018). This beneficial effect was induced by *S. cerevisiae* co-aggregation with *C. albicans*, decrease of *C. albicans* adherence to epithelial cells, and inhibition of some important virulence factors such as the pathogen’s ability to switch from bud to hyphal form. In this study, we test the effectiveness of live and inactivated *S. cerevisiae* CNCM I-3856 against OPC and examine the mechanisms of action allowing this beneficial effect.

## Materials and Methods

### *C. albicans* Strain and Culture

*C. albicans* CA1398 carrying the ACT1p-gLUC59 fusion (gLUC59) was used (Enjalbert et al., 2009). The gLUC59 luciferase reporter has previously been described (Enjalbert et al., 2009). *C. albicans* gLUC59 (BLI-*Candida*) was cultured in yeast peptone dextrose (YPD) as described by Solis and Filler (2012). The *C. albicans* CA 1398 carrying the ACT1p-gLUC59 fusion and its parental strain CA 1938 were equally pathogenic as previously demonstrated (Enjalbert et al., 2009).

### Study Products

The products studied were provided by Gnosis by Lesaffre (Marcq-en-Baroeul, France). *S. cerevisiae* live yeast (referenced as GI) is a proprietary, well-characterized strain of Lesaffre, registered in the French National Collection of Cultures of Microorganisms (CNCM) under the number I-3856. The *S. cerevisiae* species is characterized by using phenotypic (API®ID32C, Biomerieux SAS) and genotypic referenced methods (genetic amplification and sequencing of 26S DNA; Kurtzman and Robnett, 1997, 1998). Moreover, the strain CNCM I-3856 has been characterized by polymerase chain reaction (PCR) Interdelta typing techniques (European Committee for Standardization, 2009), and its genome has been sequenced. The inactivated yeast *S. cerevisiae* CNCM I-3856 (referenced as IY) is a primary grown dried whole yeast, obtained by drum drying of *S. cerevisiae* CNCM I-3856 and inactivated through the process of drying. Furthermore, the strain of *L. rhamnosus* GG (referenced as G 250) is registered in the American Type Culture Collection (ATCC) under the number 53103. The CFU counts of the probiotics used in these experiments are at least of 5 × 10^9^ CFU/g for GI and 2.5 × 10^11^ CFU/g for G 250. All products were used at 100 mg/ml.

### Mouse Model of Oropharyngeal Candidiasis

Female C57BL/6J mice from Charles River (Calco, Italy) were used at 6–8 weeks of age. Mice were treated subcutaneously with 225 mg/kg cortisone acetate (Sigma-Aldrich) every 2 days starting from 1 day before infection until the end of experiment. After anesthesia with a subcutaneous injection of a mixture of Tiletamine/Zolazepam-Xylazine (50–5 mg/kg) (Mosci et al., 2013) mice were infected with 1 × 10^6^ CFU/ml BLI-*Candida* suspension as previously described (Solis and Filler, 2012).

Then mice were treated sublingually with 10 μl of saline, FLZ (4 mg/ml), GI, IY, or G 250 (all 100 mg/ml) on days +1, +2, +3, and +6 post-infection. The oral cavity was swabbed just before the infection and streaked on YPD agar plus chloramphenicol (50 μg/ml; both from Sigma-Aldrich) to verify the absence of *Candida* spp.

### Real-Time Monitoring of Oropharyngeal Candidiasis and Pathogen Burden Determination

Starting on day 1 after challenge and at each selected day, 10 μl (0.5 mg/ml in 1:10 methanol:H_2_O) of coelenterazine (Synchem, OHM) was added sublingually. Mice were then imaged in the IVIS Lumina XRMS Imaging system (Perkin Elmer) under subcutaneous anesthesia. The total photon emission was quantified as previously described (Mosci et al., 2013). In the selected experiments, an *ex vivo* analysis of esophagus, stomach, kidneys, liver, and feces from OPC mice was performed at day +8 post-infection as previously described (Mosci et al., 2014). The *C. albicans* burden of the tongue, esophagus, stomach, and duodenum was evaluated at days +3, +6 and +8 post-infection as previously described (Mosci et al., 2014).

### Histological Analysis

The animals infected and treated with saline, FLZ, IY, GI, or G 250 were sacrificed at day +8 post-infection to analyze gross and histopathologic lesions, and tongues were excised. Histological examination was, also, performed on tongues of uninfected mice treated with saline, FLZ, GI, IY, or G 250 to evaluate the integrity of the tissue and neutrophil recruitment. The tissues were fixed immediately in 10% formalin and then embedded in paraffin. The tongues were sectioned longitudinally to evaluate the extension of the lesions. The 3–5 μm thick sections were stained using the periodic acid-Schiff (PAS) procedure to detect fungi and examined by light microscopy (Leica DM2500). The scale bars are in micrometer.

### Quantitative Analysis of ALS3, SAP2, and SAP6 Gene Expression

At day +6 post-infection tongue homogenates from mice infected and treated with saline, FLZ, GI, or IY were centrifuged at 3,000 rpm for 5 min. Then cellular fractions were lysed using Trizol reagent (Life Technology). Total RNA was extracted and retro-transcribed by using the Moloney murine leukemia virus reverse transcriptase reaction (M-MLV RT) as described in the manufacturer’s instructions. cDNA concentration was determined using a spectrophotometer. *C. albicans* ACT1, SAP2, SAP6, and ALS3 gene transcription was detected by using primers reported in the literature (Naglik et al., 2008; Roudbarmohammadi et al., 2016). Real-time quantitative PCR (RT-qPCR) was performed in 96-well PCR SYBR green plates (all from BioRad) using 200 ng of cDNA for each sample. In this way, the amount of genes expressed by *C. albicans* gLUC59 during infection was independent by fungal burden. All samples were measured in triplicate. The relative level of *Candida* gene expression was reported as 2-∆∆Ct relative to transcripts of *C. albicans* inoculum (basal gene expression level of *Candida* at day 0). Briefly, 3 days before infection, a colony of the *C. albicans* gLUC59 was added into 10 ml of YPD broth and incubate in a 30°C shaker. The next day, 100 μl of the overnight culture were transferred to 10 ml fresh YPD broth and incubate in a 30°C shaker overnight. This step was repeated one more time. The next day, *C. albicans* gLUC59 was recovered, counted, and diluted at desired concentration (Solis and Filler, 2012). Amplification conditions used were the same for ACT1, ALS3, SAP2, and SAP6 genes: 3 min at 95°C, 40 cycles of 10 s at 95°C and 30 s at primer specific annealing temperature. The experiments were performed using the Eppendorf Master cycler.

### Candidacidal Assays

At day +6 post-infection, peritoneal neutrophils of mice orally infected and treated as described in section “Materials and Methods” with saline, FLZ (4 mg/ml), GI, or IY (both at 100 mg/ml) were collected 18 h after the intraperitoneal injection of 0.5 ml endotoxin-free 10% thioglycolate solution (Difco) The percentage of neutrophils was >90% (Mosci et al., 2014). The oxidative burst of neutrophils was detected by labeling cells (4 × 10^6^/ml) with 1 μM of 2′,7′-dichlorofluoresceindiacetate (DCFH-DA) for 30 min at room temperature. Cells were then incubated with *C. albicans* (CA-6) (2 × 10^6^ CFU/ml) into a black 96-well plate (Nunc), and the emission of fluorescence was measured as previously described (Mosci et al., 2014; Ricci et al., 2019). Quantification of reactive oxygen species (ROS) production was determined by calculation of the area under curve (AUC) (Ermert et al., 2009; Kenno et al., 2016). Killing activity of neutrophils was determined by CFU assays. Briefly 0.1 ml/well of neutrophils (10^5^ cells) was immediately incubated in flat-bottom 96-well microtiter tissue culture plate with 0.1 ml/well (10^4^ cells) of *C. albicans* (CA-6) in RPMI-1640 plus 5% FCS for 2 h at 37°C plus 5% CO_2_. After incubation, plates were vigorously shaken, and cells were lysed by adding Triton X-100 (0.1% in distilled water; final concentration in the well was 0.01%). Serial dilutions were prepared in distilled water for each well. The samples were then spread on Sabouraud dextrose agar plus chloramphenicol (50 μg/ml) in triplicate, and CFU counts were determined after 24 h of incubation at 37°C. Control cultures consisted of *C. albicans* (CA-6) incubated in RPMI-1640 plus 5% FCS without effector cells. Killing activity was expressed as the percentage of CFU reduction with respect to neutrophils from uninfected mice and according to the following formula: % killing activity = 100 – (CFU experimental/CFU control) × 100.

### Statistical Analysis

GraphPad Prism 7.0 software was used for all statistical analysis presented. Data are reported as boxplot graphics with median and the 25th and 75th percentile or mean ± SEM from the experiments indicated in each figure legend. Statistical analysis was performed using Mann-Whitney *U* test. *p* < 0.05 was considered as significant.

## Results

### Effect of *S. cerevisiae* Treatment on Oropharyngeal Candidiasis

**Figure 1:**
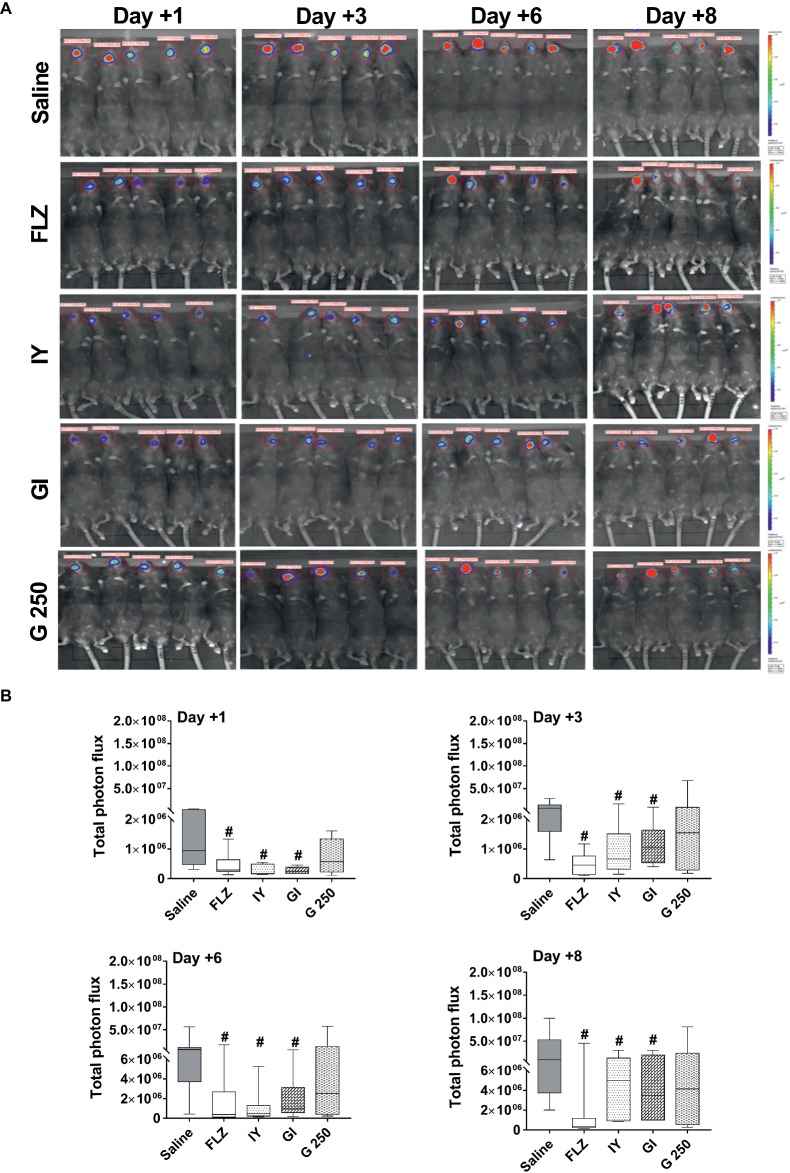
*In vivo* imaging of mice orally infected with BLI-*C. albicans* and treated with FLZ, IY, GI, or G 250. Mice were infected with BLI-*C. albicans* (1 × 10^6^ CFU/ml) and treated sublingually with 10 μl of saline, FLZ (4 mg/ml), IY, GI, or G 250 (all 100 mg/ml). At 1, 3, 6, and 8 days post-infection anesthetized mice were treated sublingually with 10 μl of coelenterazine (0.5 mg/ml) and imaged in the Lumina XRMS Imaging system. Images are representative of two separate experiments with similar results. **(A)** Total photon flux emission from oral within the images (Region Of Interest, ROI) of each mouse was quantified with Living Image R software package. **(B)** Quantification of Total photon flux emission from ROI. Data are from two different experiments each with *n* = 5 mice/group. The boxplot graphics show median including the 25th and 75th percentile. ^#^*p* < 0.05 FLZ or compound-treated infected mice vs. saline-treated infected mice.

**Figure 2:**
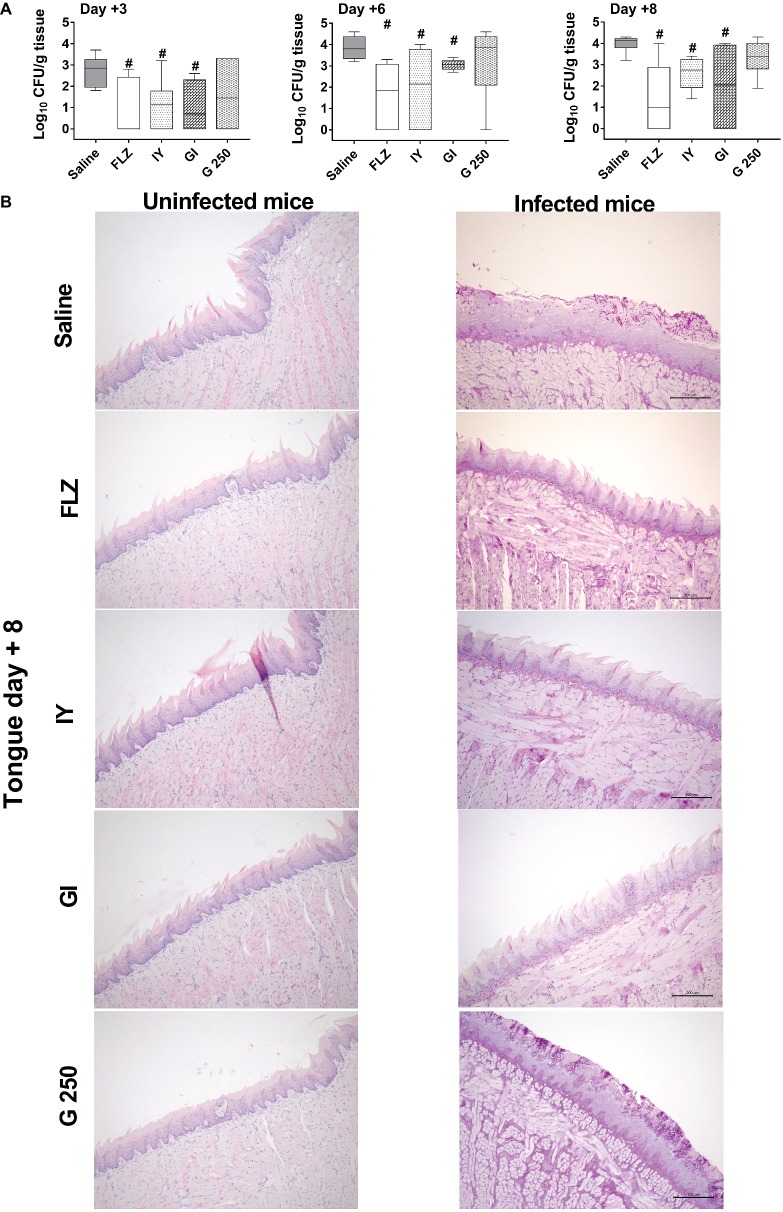
Effect of IY, GI, or G 250 treatment on tongue fungal burden and histological inflammation. **(A)** Fungal burden of mice treated with 10 μl of saline, FLZ (4 mg/ml), IY, GI, or G 250 (all 100 mg/ml) was evaluated by CFU count at days +3, +6, and +8 post-infection in tongue of infected mice. Data are from two different experiments each with *n* = 3 mice/group. The boxplot graphics show median including the 25th and 75th percentile. ^#^*p* < 0.05 FLZ or compound-treated infected mice vs. saline-treated infected mice. **(B)** Tongue sections from uninfected and infected mice treated with 10 μl of saline, FLZ (4 mg/ml), IY, GI, and G 250 (all 100 mg/ml) are shown (day +8 post-infection). Images (Bar = 200 μm, magnification 10×) are representative of two separate experiments with similar results.

To analyze whether inactivated *S. cerevisiae* (IY), live *S. cerevisiae* (GI), or live *L. rhamnosus* GG (G 250) were able to affect the course of oropharyngeal candidiasis, C57BL/6J mice were treated subcutaneously with cortisone acetate every 2 days starting from 1 day before infection until the end of the experiment. Briefly, the mice were infected with bioluminescent (BLI) *C. albicans* (1× 10^6^ CFU/ml; 10 μl/mouse) as previously described by Solis and Filler (2012). The animals were then treated sublingually with saline, FLZ (4 mg/ml; 10 μl/mouse), IY, GI, or G 250 (all 100 mg/ml; 10 μl/mouse) on days +1, +2, +3, and +6 post-infection. Saline-treated and FLZ-treated infected mice were used as negative and positive controls, respectively. The effect of the various treatments on oral candidiasis was evaluated at days +1, +3, +6, and +8 post-infection by measurement of photon emission from the oral cavity as well as by determination of CFU counts on the tongue. As shown in Figure 1, both IY and GI significantly decreased the BLI-*C. albicans* load in the oral cavity at each time point tested, both as visually evident (Figure 1A) and as measured by total photon emission (Figure 1B). Of note, the anti-*Candida* effect of IY and GI was comparable to the effect of positive control FLZ at each time point tested. By contrast, no effect was observed for G 250 (Figure 1B). The reduction of the *Candida* load in the oral cavity was also confirmed through a significant decrease in CFU counts on the tongues of IY- and GI-treated infected animals (Figure 2A). In order to characterize the lesions associated with the OPC, we performed histological analysis of the tongues at day +8 post-infection. The fungal invasion of the tongue of saline-treated infected mice was limited to a portion on the surface of the tongue due to the formation of pseudomembranes. The pathogenic fungi and the inflammatory cells appeared to be confined to the keratinized layer of the tongue where micro abscesses formation could be observed. No *Candida* cells and *Candida*-induced lesions were found in the tongues of FLZ, IY, and GI-treated infected mice (Figure 2B), while *Candida*-induced lesions were observed in the tongue of G 250-treated infected mice (Figure 2B). Histological examination of the tongues of uninfected mice treated with saline, FLZ, IY, GI, or G 250 showed complete tissue integrity and an absence of neutrophils recruitment (Figure 2B).

**Figure 3:**
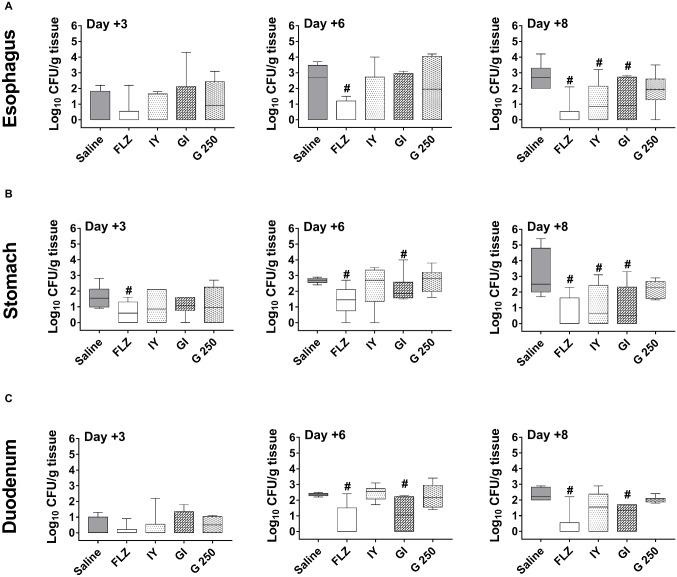
Effect of IY, GI, or G 250 treatment on target organs fungal burden. Fungal burden of infected mice treated with 10 μl of saline, FLZ (4 mg/ml), IY, GI, or G 250 (all 100 mg/ml) was evaluated by CFU count at days +3, +6, and +8 post-infection in the esophagus **(A)**, stomach **(B)**, and duodenum **(C)**. Data are from two different experiments each with *n* = 3 mice/group. The boxplot graphics show median including the 25th and 75th percentile. ^#^*p* < 0.05 FLZ or compound-treated infected mice vs. saline-treated infected mice.

**Figure 4:**
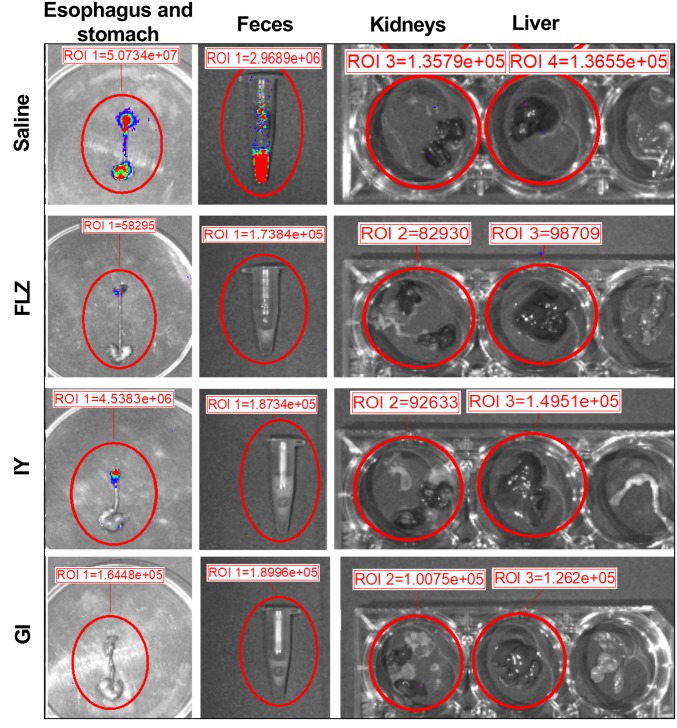
*Ex vivo* imaging of mice orally infected with BLI-*C. albicans* and treated with FLZ, IY, or GI. *Ex vivo* analysis of esophagus, stomach, feces, kidneys and liver from saline, FLZ, IY or GI-treated infected mice are shown. Briefly, 8 days post-infection mice were euthanized, and esophagus, stomach, kidneys, liver and feces were recovered and then soaked with 10 μl of coelenterazine (0.5 mg/ml) to detect the *C. albicans* burden using the Lumina XRMS Imaging system. Data are from one representative mouse from one experiment. Region Of Interest (ROI) was quantified with Living Image R software package.

The progression of the infection to esophagus, stomach, and intestine was evaluated by CFU counts at days +3, +6, and +8 post-infection. The results show that both IY and GI were able to reduce the esophageal *Candida* load only at day +8 post-infection (Figure 3A). In the stomach, the beneficial effect of GI was evident at both days +6 and +8 post-infection, while the effect of IY was appreciable only at day +8 post-infection (Figure 3B). In the duodenum, only GI induced a significant inhibition of the *C. albicans* load at days +6 and +8 post-infection (Figure 3C). By contrast, G 250 had no significant effect (Figure 3). Notably and as expected, FLZ was active (Figure 3). Given that the treatment with the *Lactobacillus* probiotic did not influence the course of oropharyngeal infection, only *S. cerevisiae* products were evaluated in the subsequent experiments. First, the effect of IY and GI was evaluated by *ex vivo* bioluminescence emission of explanted gastric tracts (pharynx, esophagus, and stomach) at day +8 post-infection. The results show that treatment with IY and GI strongly reduced the BLI signal from the explanted organs as compared to organs from saline-treated infected mice (Figure 4). Next, the BLI signal from feces was tested to examine whether IY and GI were able to accelerate the passage of *C. albicans* from the stomach to intestine. The results show that BLI-*C. albicans* was evident only in the feces of saline-treated infected mice (Figure 4). Fungus dissemination to kidneys and liver was also evaluated. No fungal load was detected in these organs in any experimental group at day +8 post-infection (Figure 4). Further observations show that, by day +8 post-infection, infected mice did not show relevant clinical signs and manifested varying extents of weight loss. G 250- and saline-treated infected mice both showed similar extent of weight loss, while the weight loss in IY- and GY- treated groups was much less pronounced (data not shown).

### Effect of *S. cerevisiae* Treatment on Hyphae-Associated Virulence Genes

**Figure 5:**
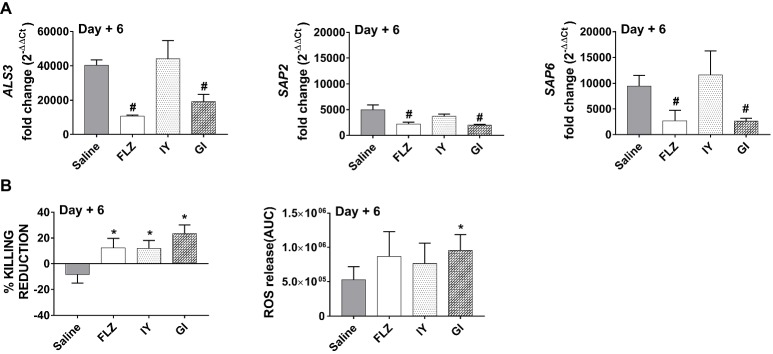
Effect of IY or GI on *C. albicans* virulence factors and on neutrophils activity. **(A)** ALS3, SAP2 and SAP6 gene expression in cellular fractions from tongue homogenates of infected mice treated with 10 μl of saline, FLZ (4 mg/ml), IY, or GI (both at 100 mg/ml) at 1, 2, and 3 days post-infection was evaluated after 6 days of infection. Tongue homogenates were centrifuged, cellular fractions were lysed, and total RNA was extracted and retro-transcribed into cDNA. ALS3, SAP2, and SAP6 genes were detected as described in section “Materials and Methods.” Data for ALS3, SAP2, and SAP6 genes are the mean ± SEM from one experiment with *n* = 3 mice/group. ^#^*p* < 0.05 FLZ or compound-treated infected mice vs. saline-treated infected mice. **(B)** Candidacidal activity of peritoneal neutrophils from infected mice, treated as above described, was evaluated after 6 days of infection. Data are the mean ± SEM from two different experiments each with *n* = 3 mice/group. *p < 0.05 FLZ or compound-treated infected mice vs. saline-treated infected mice. Reactive oxygen species (ROS) of the peritoneal neutrophils (4 × 10^6^/ml) were evaluated after 6 days of infection in the presence of *C. albicans* (2 × 10^6^ CFU/ml). Quantification of total ROS production was determined by calculation of area under curve (AUC). Data are the mean ± SEM from one experiment with *n* = 4 mice/group. *p < 0.05 FLZ or compound-treated infected mice vs. saline-treated infected mice.

In complementary experiments, we wondered if the inhibition of the pathogen load in the oral cavity may be related to the modulation of some important *C. albicans* virulence factors involved in the adhesion and invasion of epithelial cells (Naglik et al., 2011). To this end, the expression of ALS3 and two aspartyl proteases (SAP2 and 6) were analyzed. As shown in Figure 5A, the expression of the three virulence genes was significantly downregulated by the treatment with GI, but not with IY.

### Effect of *S. cerevisiae* Treatment on Neutrophil Activity

Neutrophils represent a major class of immune effector cells in fungal killing (Gazendam et al., 2016), of which reactive oxygen species production is the major killing mechanism (Moyes and Naglik, 2011), and they play a central role in protecting oral epithelium from *C. albicans* injury (Weindl et al., 2007). Given the difficulty to recover these cells from mucosal tissue, we examined whether the neutrophils recruited in the peritoneal cavity had candidacidal activity. The results reported in Figure 5B show that neutrophils from infected mice presented a reduction in killing activity as compared to those from uninfected mice and that the *in vivo* treatment with GI resulted in a greater increase in candidacidal activity of neutrophils compared to that of the two other treated groups. Moreover, the oxidative burst of neutrophils was also increased following the GI treatment. As expected, FLZ was able to increase antimicrobial capacity of neutrophils (Brummer and Stevens, 1996; Figure 5B).

## Discussion

Probiotics are defined as live microorganisms, which, when administered in appropriate amount, provide a health benefit to the host (Food and Agriculture Organization and World Health Organization Expert Consultation, 2001). The numerous mechanisms by which probiotics accomplish their beneficial actions include competitive exclusion for binding sites of pathogenic microorganisms, production of anti-microbial substances, enhancement of the epithelial barrier, and modulation of the immune system. We recently demonstrated a clear effect of some probiotics in improving the course of mucosal infections in experimental models. In particular, daily treatment with *S. cerevisiae* CNCM I-3856 resulted in curing bacterial vaginosis (Pericolini et al., 2017; Sabbatini et al., 2018) and vaginal candidiasis in infected mice (Gabrielli et al., 2018). Here we demonstrated that the administration of live (GI) or inactivated (IY) *S. cerevisiae* CNCM I-3856 in the oral cavity results in a protective effect against oral candidiasis. A complementary set of experiments allows us to demonstrate that this protective effect is related to: (1) a significant inhibition of the *C. albicans* load in the oral cavity as well as in the tongue that showed an early resolution of the inflammatory process and (2) a progressive reduction of the pathogen load measured in the esophagus, stomach, and duodenum resulting in a clearance of *C. albicans* from the intestine. The marked increase in the antimicrobial capacity of neutrophils was related to GI upregulation of oxygen-dependent mechanisms. GI was, also, able to inhibit the expression of some *Candida* virulence factors, which is not verified for IY. Although the two yeast products have shown beneficial effects, these appeared to be more significant with live yeast. This is consistent with previous data showing that viable bacteria are more effective than non-viable bacteria for health benefit (Lahtinen, 2012).

Previous studies demonstrate that *L. rhamnosus* is able to interfere with *C. albicans* growth in *in vitro* experimental model (Allonsius et al., 2017). Our data show that *L. rhamnosus* GG was ineffective in our *in vivo* model of oral candidiasis, and this apparent discrepancy may be due to different experimental models used. Indeed, the effect of *L. rhamnosus* against candidiasis is hitherto unsubstantiated with *in vivo* evidence.

Despite the growing favor for the use of probiotics for oral infections (Cagetti et al., 2013; Laleman et al., 2014; Gruner et al., 2016), only few studies focusing on probiotic bacteria (*lactobacilli* and *bifidobacteria*) are available. These studies target specifically the elderly (Ai et al., 2017). None of the studies investigated yeast strains.

In this study, and for the first time, we provide evidence for the efficacy of a probiotic yeast in accelerating the clearance of *C. albicans* in an experimental model of OPC in mice. Furthermore, the daily administration of *S. cerevisiae* CNCM I-3856 resulted in a drastic reduction of pathogenic fungal load that is also related to the early quenching of the inflammatory process in the tongue. This effect was also observed when inactivated yeast was used instead of live yeast. Given that *S. cerevisiae* and *C. albicans* share some adhesins, it is likely that *S. cerevisiae* compete with the attachment of *C. albicans* (Tada et al., 2002) to epithelial cells, thus inhibiting the colonization and dissemination of the pathogen. This can account for the complete resolution of the inflammatory process in the tongue of GI- and IY-treated infected mice.

*C. albicans* from the oral cavity (Hisajima et al., 2008; Patil et al., 2015) can spread to the pharynx and/or esophagus, stomach, and intestine (Mosci et al., 2013). In our experimental model, *C. albicans* cells were detected in the esophagus, stomach, and duodenum. The fungal load was progressively reduced after treatment with GI to the same extent as that observed with the conventional antifungal treatment (FLZ). In particular, no significant effect from GI and IY treatment was observed early on the infection time course (up to day +3 post-infection), while the beneficial effect of GI and IY was clearly noted later (from day +8 post-infection onward).

Late after the infection (on day +8 post-infection), *C. albicans* was recovered from the feces, suggesting the passage of *Candida* from the stomach to the gut without treatment (Mosci et al., 2013; Prieto and Pla, 2015). It is noteworthy that no such presence was observed in the mice treated with GI, IY, and FLZ, suggesting that the consistent reduction of *C. albicans* growth in various organs can prevent the intestine from being colonized/invaded by the pathogen. This is also relevant considering that the presence of *C. albicans* in the intestine is associated with different types of pathological conditions (Standaert-Vitse et al., 2009; Sokol et al., 2017).

Indeed, GI is able to reduce the expression of adhesion molecules such as ALS3 (Liu and Filler, 2011), and this is consistent with previously observed inhibition of the pathogen adhesion to epithelial cells (Pericolini et al., 2017; Gabrielli et al., 2018). In addition, GI treatment resulted in an impairment of the transition of *C. albicans* to mycelial form as well as of the expression of aspartyl proteases SAP2 and SAP6, both of which are highly involved in the colonization and dissemination of *C. albicans* (Naglik et al., 2003, 2004). In contrast to GI, IY is ineffective in regulating the expression of *C. albicans* virulence factors and yeast-to-hyphae transition. This may account for the lower protective effect obtained with IY.

Additional evidence of GI mediated effects is provided through the increased antimicrobial capacity of neutrophils related to an increase of oxygen-mediated mechanisms suggesting ROS-driven candidacidal activity.

Collectively, our results show that live and inactivated *S. cerevisiae* CNCM I-3856 is able to strongly reduce the local fungal burden commonly observed in OPC in the oral cavity, the esophagus, and the stomach, thus preventing the translocation of *C. albicans* to the small intestine. The inactivated yeast showed an inferior protective effect as compared to the live probiotic yeast. Furthermore, *L. rhamnosus* GG was completely ineffective. These results suggest that probiotic *S. cerevisiae* CNCM I-3856 is able to positively reverse/attenuate the course of OPC infection.

## Data Availability

The raw data supporting the conclusions of this manuscript will be made available by the authors, without under reservation, to any qualified researcher.

## Ethics Statement

All animal experiments were performed in agreement with the EU Directive 2010/63, the European Convention for the Protection of Vertebrate Animals used for Experimental and other Scientific Purposes, and the National Law 116/92. The protocol was approved by the Perugia University Ethics Committee and by the Modena and Reggio Emilia University Ethics Committee for animal care and use (Comitato Universitario di Bioetica and Organismo Preposto al Benessere degli Animali, permit number 223/2016-PR). The animals used for the real-time monitoring of oropharyngeal candidiasis were housed in the animal facility of the University of Modena and Reggio Emilia (Centro Servizi Stabulario Interdipartimentale, BIOSTAB, Authorization number 268/2011-A), and the animals used for all the other experiments were housed in the animal facility of the University of Perugia (Authorization number 34/2003A).

## Author Contributions

AV, NB, and CM conceived the study. AV, CM, and NB developed the project and designed the research. ER, SS, PM, and EP performed the experiments. AV, CM, and NB wrote the manuscript. EB, SP, AD, AV, CM, and NB analyzed and discussed the data. All authors contributed to the writing of the statement, agreed with its content and conclusions, and read and approved the final manuscript.

### Conflict of Interest Statement

NB and AD were employed by the company Lesaffre International, Lesaffre Group, Marcq-en-Baroeul, France.

The remaining authors declare that the research was conducted in the absence of any commercial or financial relationships that could be construed as a potential conflict of interest.
